# Announcement

**DOI:** 10.1155/1994/85049

**Published:** 1994


					ANNOUNCEMENT
The 2nd Alps-Adria Congress on Hepato-Pancreato-Biliary Surgery and Medicine
will be held in Pavia (Italy) on September 13-16, 1995, under the auspices of the new International
HPB Association.
For further information, please contact:
Professor Eugenio Forni
Clinica Chirurgica Generate
Universita degli Studi di Pavia
27100 Pavia
Italy
Tel: (+39) 382 526216
Fax: (+39) 382 528325

				

